# Efficacy and Tolerability of Erlotinib 100 mg/d vs. Gefitinib 250 mg/d in EGFR-Mutated Advanced Non-small Cell Lung Cancer (E100VG250): An Open-Label, Randomized, Phase 2 Study

**DOI:** 10.3389/fonc.2020.587849

**Published:** 2020-11-10

**Authors:** Shen Zhao, Zhen Zhang, Wenfeng Fang, Yaxiong Zhang, Zhonghan Zhang, Shaodong Hong, Yuxiang Ma, Ting Zhou, Yunpeng Yang, Yan Huang, Hongyun Zhao, Li Zhang

**Affiliations:** ^1^Department of Medical Oncology, Sun Yat-sen University Cancer Center, Guangzhou, China; ^2^State Key Laboratory of Oncology in South China, Guangzhou, China; ^3^Collaborative Innovation Center for Cancer Medicine, Guangzhou, China; ^4^Zhongshan School of Medicine, Sun Yat-sen University, Guangzhou, China; ^5^Department of Clinical Research, Sun Yat-sen University Cancer Center, Guangzhou, China

**Keywords:** non-small cell lung cancer, randomized controlled trial, lower dose, erlotinib, gefitinib, EGFR mutation

## Abstract

**Background:** Erlotinib-based combination therapy leads to increased efficacy but also toxicity for EGFR-mutated NSCLC. Reducing the dose of erlotinib could improve treatment tolerability, but few evidences are available regarding its efficacy at reduced dose. This randomized phase-2 study intends to compare the efficacy and tolerability between lower dose erlotinib (100 mg/d) and standard dose gefitinib (250 mg/d) in EGFR-mutated NSCLC.

**Methods:** Patients with EGFR-mutated advanced NSCLC were randomized at 1:1 ratio to receive erlotinib 100 mg/d or gefitinib 250 mg/d until disease progression or unacceptable toxicity. The primary endpoint was disease control rate (DCR).

**Results:** Between April 2013 and September 2018, 171 patients were randomized to receive erlotinib (*n* = 85) and gefitinib (*n* = 86); 74 in the erlotinib group and 83 in the gefitinib group were include in analysis. DCR with erlotinib and gefitinib were 91% [95% CI 81.7–95.3] and 93% [85.1–96.6], respectively (*P* = 0.613). Response rate was 62% [50.8–72.4] in the erlotinib group and 53% [42.4–63.4] in the gefitinib group (*P* = 0.247). No significant difference was observed between erlotinib and gefitinib in median progression-free survival [10.1 vs. 11.3 months, HR = 1.295 [0.893–1.879], *P* = 0.171] and median overall survival [26.6 vs. 28.7 months, HR = 0.999 [0.637–1.569], *P* = 0.998]. Subgroup analyses by line of treatment, EGFR subtypes and status of central nervous system (CNS) metastasis found similar results. More toxicity [any-grade, 80 [96%] vs. 66 [89]; grade 3–4, 11 [13%] vs. 4 [5%]] and toxicity-related discontinuation [10 [12%] vs. 3 [4%]] occurred with gefitinib compared with erlotinib. But no significant difference was observed.

**Conclusion:** Lower dose erlotinib (100 mg/d) achieved comparable efficacy compared with standard dose gefitinib (250 mg/d) in EGFR-mutated NSCLC.

**Clinical Trial Registration:** https://clinicaltrials.gov, identifier: NCT01955421.

## Introduction

Epidermal growth factor receptor (EGFR) tyrosine kinase inhibitors (TKIs) are standard first-line treatment for EGFR mutation-driven non-small cell lung cancer (NSCLC) (1). Median progression-free survival (PFS) was 10–13 months with first-generation TKIs (gefitinib and erlotinib), 14.7 months with second-generation TKI (dacomitinib), and 18.9 months with third-generation agent (osimertinib) (2–7). Given that most EGFR-driven NSCLC patients fail to benefit from the recent advance in immunotherapy, treatment options after the exhaustion of targeted therapy are highly limited (8). Therefore, it remains a crucial need to develop EGFR TKI-based combination therapies that can optimize tumor control and delay disease progression (9–11). In the recent RELAY trial, the combination of erlotinib and ramucirumab yielded an unprecedented median PFS of 19.4 months, accompanied by a 72% incidence of grade 3–4 treatment-related adverse events (10).

Reducing the dose of erlotinib, which is now approved at its maximal tolerated dose (MTD) 150 mg/d (12), may improve the tolerability of combination therapy. However, data regarding the efficacy of lower dose erlotinib are limited and mutually contradictory. Preclinical models and phase-1 pharmacokinetic data suggested that erlotinib 25 mg/d led to similar antitumor effect compared with gefitinib 250 mg/d (12, 13). Retrospective studies also supported this notion by showing similar PFS between patients treated with reduced-dose erlotinib (≤ 100 mg/d) and those with standard dose (14). *Post-hoc* analyses that might be subjected to survival bias found a correlation between dose reduction of EGFR TKIs and better treatment outcomes (15, 16). A single-arm phase-2 trial showed 50 mg/d erlotinib achieved an objective response rate of 60% in elderly or frail patients (17). Nevertheless, another single-arm, prospective study reported contradictory findings, where no objective response was observed in patients treated with erlotinib 50 mg/d (18).

There has been no prospective, randomized controlled trial (RCT) directly comparing lower dose erlotinib with standard dose erlotinib or gefitinib in EGFR-mutated advanced NSCLC. Therefore, to properly addressed this problem, we designed this randomized, phase-2 study comparing the efficacy and tolerability of erlotinib 100 mg/d vs. gefitinib 250 mg/d in patients with EGFR-mutated advanced NSCLC.

## Patients and Methods

### Study Design and Patients

This is an open-label, randomized, phase-2 study to compare the efficacy and tolerability of erlotinib 100 mg/d vs. gefitinib 250 mg/d in patients with EGFR-mutated, advanced NSCLC.

Eligibility criteria were aged at least 18 years; histologically or cytologically confirmed stage IIIB/IV NSCLC defined by American Joint Committee on Cancer (AJCC) staging criteria (version 7); stage IIIB had no indication for curative treatment; harbored EGFR exon 19 or 21 sensitizing mutations detected by direct sequencing or Amplification Refractory Mutation System (ARMS); measurable disease according to Response Evaluation Criteria in Solid Tumors (RECIST) version 1.1 (19); an Eastern Cooperative Oncology Group (ECOG) performance status of 0 to 2; adequate bone marrow, liver and kidney function; no prior exposure to EGFR TKIs, able to swallow tablets and resolution to grade 1 or less adverse events due to any previous anticancer treatment. Patients with EGFR T790M mutations, clinically unstable CNS metastasis (symptomatic, or needed treatment within 4 weeks, or pia mater disease), clinically relevant cardiovascular diseases, history of interstitial lung diseases, other active malignancies or active infectious diseases were excluded.

Study protocol was approved by the ethics committee of Sun Yat-sen University Cancer Center. All patients had provided written informed consent before the study entry. The study was conducted in accordance with the Declaration of Helsinki and the International Conference on Harmonization guidelines for good clinical practice.

### Procedures

Eligible patients were randomly assigned to received erlotinib 100 mg/d or gefitinib 250 mg/d at a 1:1 ratio using an interactive web-response system with a computer-generated random sequence. Patients and investigators were all unmasked to treatment allocation. Treatment could be delayed for up to 2 weeks for recovery from toxicities, and was reintroduced at the same dosage when recover to grade 1 or baseline. Dose modification of was not allowed. Treatment continued until radiographic progression according to RECIST version 1.1, or intolerable toxicity or withdrawal of consent.

Baseline CT scans and brain MRI were mandated for every patient. Tumor assessment by CT scans were performed 4 weeks after randomization, and every 8 weeks after the first assessment. For patients with baseline CNS metastasis, CT scans, and brain MRI were both performed for every assessment. Tumor responses were evaluated by investigators according to RECIST version 1.1. Patients were evaluated for adverse events at every visit. Adverse events were graded according to the National Cancer Institute Common Terminology Criteria for Adverse Events version 4.0. Treatment adherence was monitored by monthly telephone follow-up.

### Outcomes

The primary endpoint was disease control rate (DCR) in the full-analysis set, defined as the sum proportion of patients achieving complete responses, or partial responses or stable diseases according to RECIST version 1.1. Secondary endpoints included objective response rate (ORR, the sum proportion of patients achieving complete responses or partial responses), PFS (the time from randomization to disease progression or death from any cause), and overall survival (OS, the time from randomization to date of death from any cause). Prespecified subgroup analyses were planned to evaluate efficacy of erlotinib 100 mg/d in treatment-naïve patients, patients with different EGFR mutations (exon 19 deletions, L858R mutations), and patients with or without baseline CNS metastasis.

### Statistical Analysis

This randomized phase-2 trial was designed to investigate the efficacy and tolerability of erlotinib at 100 mg/d compared with gefitinib at 250 mg/d and determine whether it will be useful to proceed to a phase-3 non-inferiority trial. The criteria for proceeding to a phase-3 non-inferiority trial should be that the lower limit of the 95% CI on difference in DCR (i.e., the lower 95% CI for the DCR of erlotinib group minus DCR of gefitinib group) was not more than 12%.We estimated a DCR of 91% for gefitinib 250 mg/d based on the data from WJTOG3405 and ICOGEN (3, 20). Therefore, comparable efficacy could be concluded and a phase-3 non-inferiority trial was warranted if the lower limit of the 95% CI of DCR with erlotinib 100 mg/d was > 79%. At least 71 patients are required in each group to draw a useful conclusion with an 80% statistical power at a two-sided significance level of 5%. Assuming a 12% dropout rate, the estimated sample size was set at 160 patients with 80 patients for each group.

Patient characteristics, tumor responses, and adverse events were compared between the two groups using the χ^2^ or Fisher's exact test. Survival was estimated using the Kaplan-Meier method. A two-sided log-rank test was used to compare survival between two treatment groups. Estimates of the treatment effect on survival were summarized as a hazard ratio (HR) for erlotinib vs. gefitinib with a two-sided 95% confidence interval (CI). HR and the corresponding 95% CI were calculated with the Cox proportional hazards regression model. All *P-*values were two-sided. Analyses were conducted using SAS, version 9.3 (SAS Institute, Cary, NC). This study is registered at ClinicalTrials.gov as NCT01955421.

## Results

### Patients Characteristics

**Figure 1:**
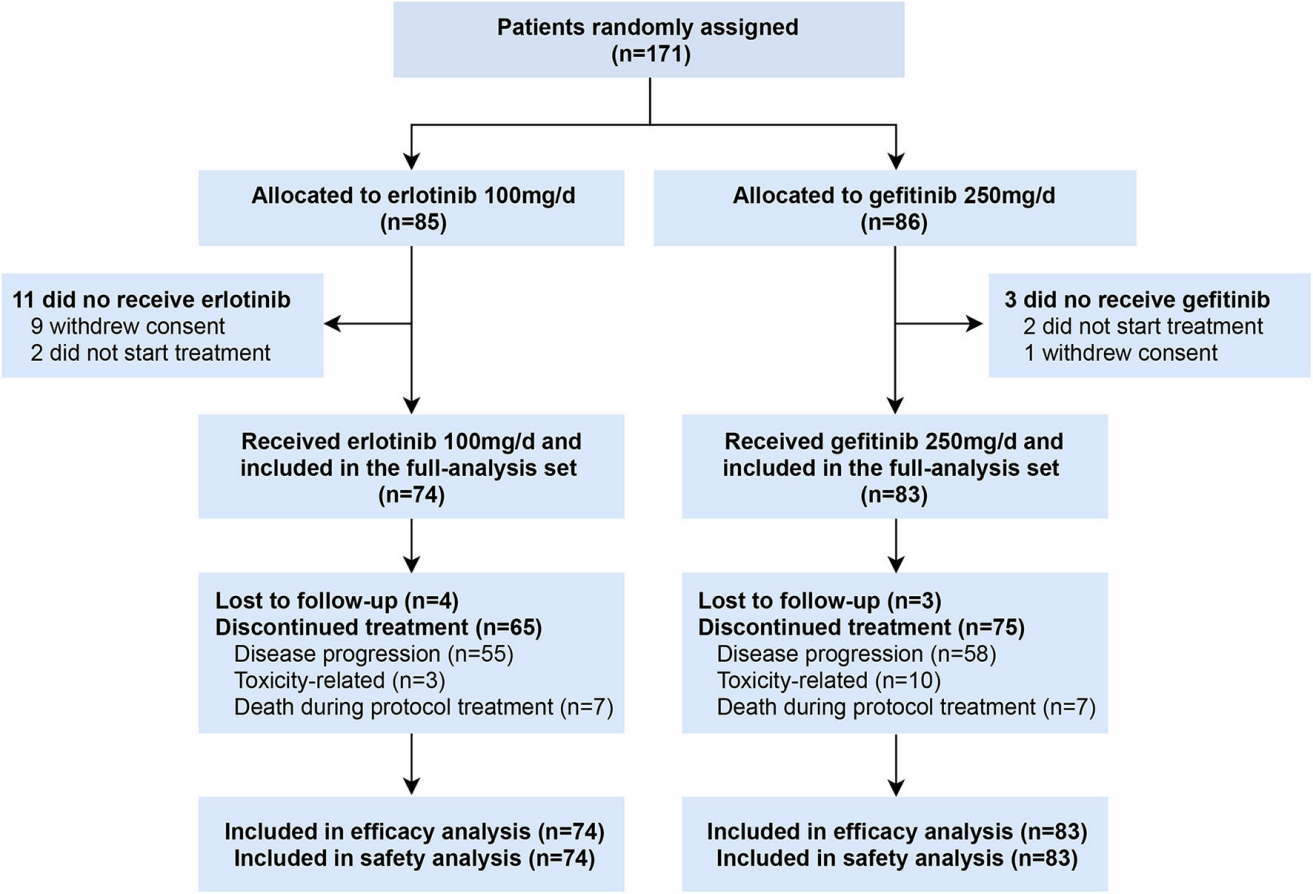
CONSORT flow diagram (Data cutoff date: Sept 30, 2019).

Between April 2013 and September 2018, 171 patients were enrolled, of whom 85 were randomly assigned to receive erlotinib 100 mg/d and 86 to gefitinib 250 mg/d. Ten patients withdrew before initiation of treatment and four patients received other EGFR inhibitors (2 afatinib, 2 icotinib) (Figure 1). A total of 157 patients who received at least one dose of investigated drugs were included in the analysis population (full-analysis set: 74 erlotinib, 83 gefitinib). Baseline demographic and clinicopathological characteristics of patients were balanced between two groups (Table 1). Most patients with baseline brain metastasis were asymptomatic and untreated (20 erlotinib, 25 gefitinib). Thirteen patients received brain radiotherapy (8 erlotinib, 5 gefitinib) and one patient in the erlotinib group received surgical resection of brain metastasis before enrollment.

**Table 1:** Baseline patient characteristics of the study.

**Patient characteristics**	**No. of patients (%)**			***P***
	**Erlotinib (*n =* 74)**	**Gefitinib (*n =* 83)**	**All patients (*n =* 157)**	
**Age, years**				0.934
Median (range)	57 (27–77)	56 (32–82)	56 (27–82)	
<65	61 (82)	68 (82)	129 (82)	
≥ 65	13 (18)	15 (18)	28 (18)	
**Sex**				0.256
Male	37 (50)	34 (41)	71 (45)	
Female	37 (50)	49 (59)	86 (55)	
**ECOG PS**				0.422
0–1	70 (95)	81 (98)	151 (96)	
2	4 (5)	2 (2)	6 (4)	
**Histology**				0.851
Adeno	69 (93)	78 (94)	147 (94)	
Non-adeno^a^	5 (7)	5 (6)	10 (6)	
**Disease stage**				0.602
IV	72 (97)	82 (99)	154 (98)	
IIIB	2 (3)	1 (1)	3 (2)	
**Line of EGFR-TKI**				0.774
1st line	55 (74)	60 (72)	115 (73)	
2nd line or beyond^b^	19 (26)	23 (28)	42 (27)	
**Prior Surgery**				0.828
Yes	15 (20)	18 (22)	33 (21)	
No	59 (80)	65 (78)	124 (79)	
**Prior Radiotherapy**				0.736
Yes	5 (7)	4 (5)	9 (6)	
No	69 (93)	79 (95)	148 (94)	
**Prior Chemotherapy**^c^				0.774
Yes	19 (26)	23 (28)	42 (27)	
No	55 (74)	60 (72)	115 (73)	
**Baseline CNS metastasis**				0.694
Yes	29 (39)	30 (36)	59 (38)	
No	45 (61)	53 (64)	98 (62)	
**Baseline Liver metastasis**				0.316
Yes	13 (18)	20 (24)	33 (21)	
No	61 (82)	63 (76)	124 (79)	
**EGFR mutation**				0.593
Exon19 deletion	46 (62)	45 (54)	91 (58)	
L858R mutation	27 (36)	36 (43)	63 (40)	
Others^d^	1 (1)	2 (2)	3 (2)	

ECOG PS, Eastern Cooperative Oncology Group performance status; Adeno, adenocarcinoma; EGFR TKI, epidermal growth factor receptors tyrosine kinase inhibitors; EGFR, epidermal growth factor receptors.

a non-adenocarcinoma included squamous-cell carcinoma (n = 6), large-cell carcinoma (n = 3), and bronchoalveolar carcinoma (n = 1).

b included four patients in the third-line settings.

c patients who had received chemotherapy were all treated with at least one cycle of platinum-based doublet chemotherapy.

d *included two patients with L861Q mutations, one patient with G719A mutation*.

### Response and Survival

The data cutoff date was September 30, 2019, when 113 progression events had occurred. Median follow-up was 21.4 months (Interquartile range: 12.7–28.6).

**Figure 2:**
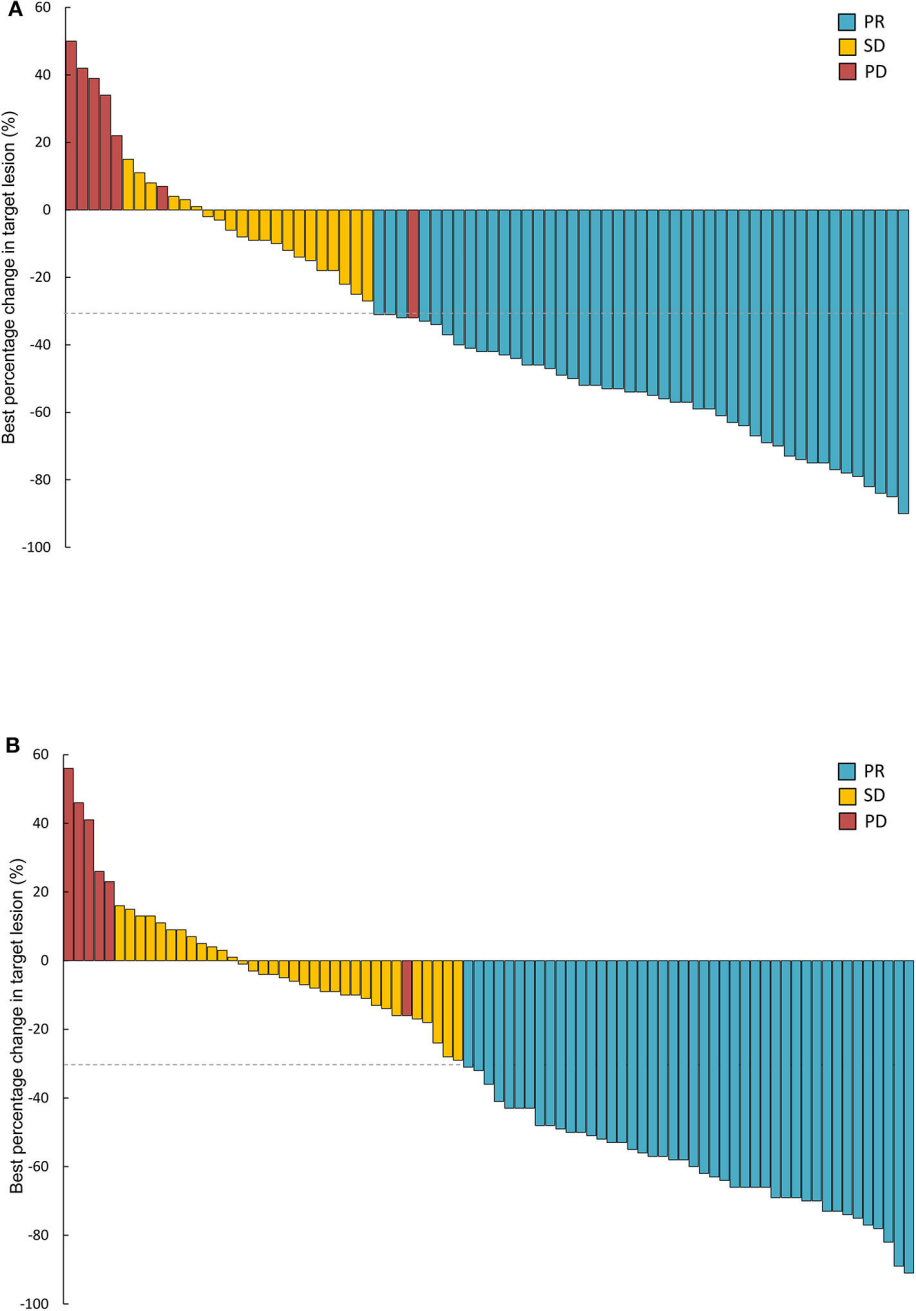
Waterfall plots of best percentage changes in the target lesions at baseline in two groups. **(A)** Erlotinib 100 mg/d group (*n* = 74). **(B)** Gefitinib 250 mg/d group (*n* = 83). PR, partial response; SD, stable disease; PD, progressive disease.

Treatment responses of the full-analysis set and subgroup population are presented in Table 2. Best percentage changes in the target lesion for two groups are shown in Figure 2. The proportion of patients achieved disease control with erlotinib 100 mg/d was similar to those with standard dose gefitinib [91% [95%CI 81.7–95.3] vs. 93% [95%CI 85.1–96.6], *P* = 0.613, Table 2]. The difference in DCR between erlotinib and gefitinib group was 2% and the lower 95% CI for the difference in DCR was 11.3%. Therefore, the primary endpoint of this study was met. Forty six patients [62% [95%CI 50.8–72.4]] in the erlotinib group and 44 patients [53% [95%CI 42.4–63.4]] in the gefitinib group had an objective response, respectively (*P* = 0.247). Median time to response was also similar between erlotinib and gefitinib [29 days [95% CI 26–63] vs. 32 days [95% CI 28–85], *P* = 0.142]. However, median duration of response with erlotinib 100 mg/d was significantly shorter than with standard dose gefitinib [7.7 months [95% CI 6.1–10.1] vs. 10.6 months [95% CI 6.3–12.9], *P* = 0.020]. Subgroup analyses were performed by the line of treatment, mutation subtypes and status of CNS metastasis. In terms of DCR and ORR, no significant difference was observed between lower dose erlotinib and standard dose gefitinib in subgroup populations (Table 2).

**Table 2:** Response to treatment by RECIST 1.1.

**Best Response**	**No. of Patients (%)**											
	**Full-analysis set**		**Treatment-naïve**		**Exon19 deletion**		**L858R mutation**		**With baseline CNS metastasis**		**No. baseline CNS metastasis**	
	**Erlotinib (*n =* 74)**	**Gefitinib (*n =* 83)**	**Erlotinib (*n =* 55)**	**Gefitinib (*n =* 60)**	**Erlotinib (*n =* 46)**	**Gefitinib (*n =* 45)**	**Erlotinib (*n =* 27)**	**Gefitinib (*n =* 36)**	**Erlotinib (*n =* 29)**	**Gefitinib (*n =* 30)**	**Erlotinib (*n =* 45)**	**Gefitinib (*n =* 53)**
Partial response	46 (62)	44 (53)	38 (69)	33 (55)	31 (67)	26 (58)	15 (56)	17 (47)	16 (55)	13 (43)	30 (67)	31 (58)
Stable disease	21 (28)	33 (40)	14 (25)	23 (38)	13 (28)	18 (40)	8 (30)	15 (42)	10 (34)	14 (47)	11 (24)	19 (36)
Progressive disease	7 (9)	6 (7)	3 (5)	4 (7)	2 (4)	1 (2)	4 (15)	4 (11)	3 (10)	3 (10)	4 (9)	3 (6)
**ORR**	46 (62)	44 (53)	38 (69)	33 (55)	31 (67)	26 (58)	15 (56)	17 (47)	16 (55)	13 (43)	30 (67)	31 (58)
95% CI	(50.8–72.4)	(42.4–63.4)	(56.0–79.7)	(42.5–66.9)	(53.0–79.1)	(43.3–71.0)	(37.3–72.4)	(32.0–63.0)	(37.5–71.6)	(27.3–60.8)	(52.1–78.6)	(45.1–70.7)
*P*-value	0.247		0.120		0.343		0.513		0.363		0.405	
**DCR**	67 (91)	77 (93)	52 (95)	56 (93)	44 (96)	44 (98)	23 (85)	32 (89)	26 (90)	27 (90)	41 (91)	50 (94)
95% CI	(81.7–95.3)	(85.1–96.6)	(85.1–98.1)	(84.1–97.4)	(85.5–98.8)	(88.4–99.6)	(67.5–94.1)	(74.7–95.6)	(73.6–96.4)	(74.4–96.5)	(79.3–96.5)	(84.6–98.1)
*P*-value	0.613		1.000		1.000		0.715		1.000		0.670	

*95% CI, 95% confidence interval; ORR, objective response rate; DCR, disease control rate*.

**Figure 3:**
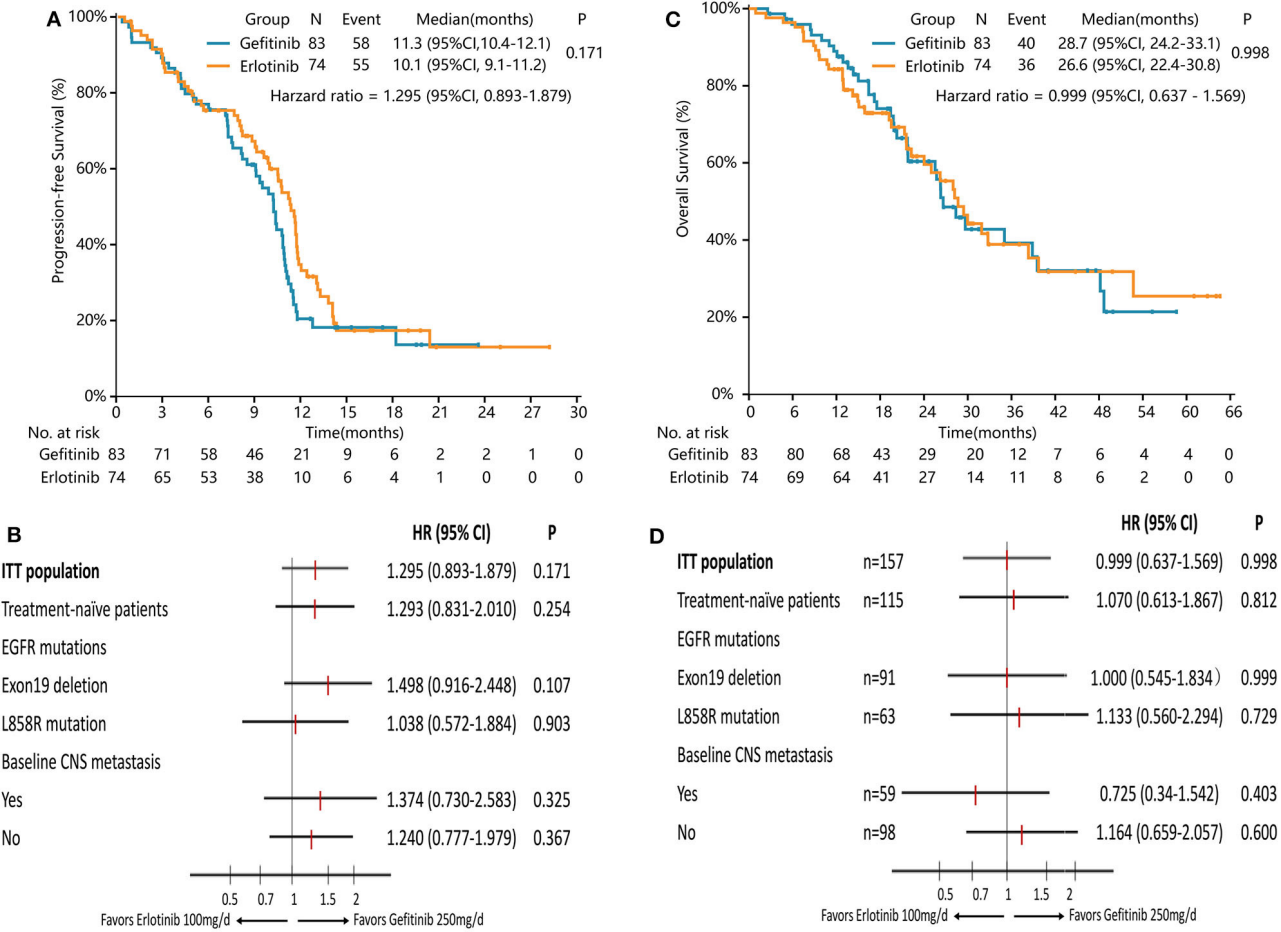
Survival analysis in the full-analysis set and subgroups by clinical characteristics. **(A)** Kaplan-Meier curves of PFS in the full-analysis set. **(B)** Subgroup analysis of PFS by the line of treatment, EGFR mutation types, and baseline CNS metastasis. **(C)** Kaplan-Meier curves of OS in the full-analysis set. **(D)** Subgroup analysis of OS by the line of treatment, EGFR mutation types, and baseline CNS metastasis.

PFS was similar between erlotinib and gefitinib [10.1 months [95% CI 9.1–11.2] vs. 11.3 months [95% CI 10.4–12.1], HR = 1.295 [95% CI 0.893–1.879], *P* = 0.171, Figure 3A]. Subgroup analyses by line of treatment, mutation subtypes, and status of CNS metastasis detected no significant difference in PFS between the two groups (Figure 3B). With regard to the patterns of disease progression, 39 patients (39/55, 71%) with lower dose erlotinib and 36 patients (36/58, 62%) with standard dose gefitinib experienced disease progression at all sites, respectively (*P* = 0.320). Twenty-one patients (21/55, 38%) with erlotinib and 31 patients (31/58, 53%) with gefitinib had disease progression in the CNS (*P* = 0.104). Among them, six patients (6/55, 11%) with lower dose erlotinib developed newly onset brain metastasis, while 14 patients (14/58, 24%) with standard dose gefitinib had newly onset brain metastasis (*P* = 0.066).

Median OS with lower dose erlotinib was numerically shorter than standard dose gefitinib, but the difference was not significant [26.6 months [95% CI 22.4–30.8] vs. 28.7 months [95% CI 24.2–33.1], HR = 0.999 [95% CI 0.637–1.569], *P* = 0.998, Figure 3C]. Subgroup analyses by line of treatment, mutation subtypes, and status of CNS metastasis showed no significant difference in OS between the two groups either (Figure 3D).

### Treatment-Related Toxicity

Toxicity was evaluable in 157 patients (Table 3). The most common treatment-related toxicity was skin and mucosa disorder, including rash, pruritus, stomatitis, and paronychia. Grade 1–2 liver dysfunction and diarrhea were also common in both groups.

**Table 3:** Treatment-related adverse events.

**Adverse event**	**No. of Patients (%)**							
	**Erlotinib (*****n*** **=** **74)**				**Gefitinib (*****n*** **=83)**			
	**All grade**	**Grade 1–2**	**Grade 3**	**Grade 4**	**All grade**	**Grade 1–2**	**Grade 3**	**Grade 4**
Rash	35 (47)	32 (43)	2 (3)	1 (1)	33 (40)	31 (37)	2 (2)	0
Diarrhea	12 (16)	12 (16)	0	0	16 (19)	15 (18)	1 (1)	0
Pruritus	9 (12)	9 (12)	0	0	15 (18)	15 (18)	0	0
Stomatitis	6 (8)	6 (8)	0	0	8 (10)	8 (10)	0	0
Increased ALT	15 (20)	14 (19)	1 (1)	0	22 (27)	16 (19)	5 (6)	1 (1)
Increased AST	11 (15)	11 (15)	0	0	21 (25)	17 (20)	3 (4)	1 (1)
Neutropenia	3 (4)	3 (4)	0	0	1 (1)	1 (1)	0	0
Increase bilirubin	3 (4)	3 (4)	0	0	8 (10)	8 (10)	0	0
Paronychia	2 (3)	2 (3)	0	0	3 (4)	3 (4)	0	0
Fatigue	1 (1)	1 (1)	0	0	2 (2)	2 (2)	0	0
Nausea/vomiting	1 (1)	1 (1)	0	0	4 (5)	4 (5)	0	0
Infection	1 (1)	1 (1)	0	0	1 (1)	1 (1)	0	0

*ALT, alanine aminotransferase; AST, aspartate aminotransferase*.

No significant difference was observed in the incidence of adverse events of any grade or adverse events of grade 3–4 between erlotinib and gefitinib. Numerically, higher incidence of alanine transaminase (ALT) and aspartate transaminase (AST) elevation was observed in the gefitinib group, but the difference was not significant [ALT: 22 [27%] vs. 15 [20%], *P* = 0.358; AST: 21 [25%] vs. 11 [15%], *P* = 0.105]. Numbers of patients with adverse events of any grade and adverse events of grade 3–4 were also higher with standard dose gefitinib compared with lower dose erlotinib [gefitinib vs. erlotinib: any-grade, 80 [96%] vs. 66 [89]; grade 3–4, 11 [13%] vs. 4 [5%]]. In the erlotinib group, three patient discontinued treatment because of serious skin toxicities. In the gefitinib group, 10 patients discontinued treatment because of grade-3 liver dysfunction (*n* = 7), grade-3 rash (*n* = 2), or grade-2 stomatitis (*n* = 1). No significant difference in toxicity-related treatment discontinuation between the two groups (*P* = 0.085). No treatment-related death occurred.

## Discussion

This is the first randomized controlled trial to directly compare lower dose erlotinib with standard dose gefitinib in EGFR-mutated NSCLC. The study objective was to evaluate whether erlotinib administered at 100 mg/d, two-thirds of its approved dose, could deliver similar efficacy compared with gefitinib 250 mg/d. According to our results, the lower 95% CI difference in DCR was <12%, indicating the need in proceeding to a phase-3 non-inferiority trial. Erlotinib 100 mg/d was comparable to gefitinib 250 mg/d in terms of disease control, tumor response, PFS, OS, and toxicity, supporting the use of 100 mg/d erlotinib in patients with EGFR-mutated, advanced NSCLC.

Erlotinib and gefitinib are both first-generation EGFR TKI. Gefitinib was administered at 250 mg/d, almost one-third of its MTD, while erlotinib was administered exactly at its MTD, 150 mg/d (12, 21, 22). Several retrospective studies have reported that dose reduction of erlotinib correlated with better response and longer survival (13, 15, 16). However, restricted by the inherent limitations of retrospective analysis, no study could provide conclusive evidence on the efficacy of reduced dose erlotinib. Additionally, given the 3–6% cerebrospinal fluid penetration rates of erlotinib and its active metabolite (23, 24), the concern that dose reduction may result in higher rate of CNS failure further discourage the use of lower dose erlotinib. In the full-analysis set of the present study, efficacy with erlotinib 100 mg/d was comparable to those with gefitinib 250 mg/d. Subgroup analysis in patients with baseline CNS metastasis [29 [39%] in erlotinib group, 30 [36%] in gefitinib group] also found that disease control, tumor response, PFS, and OS with erlotinib 100 mg/d were similar to those with gefitinib 250 mg/d. These results suggest that pharmacokinetic factor may not be the main reason for CNS failure in these patients. Erlotinib 100 mg/d is of sufficient efficacy for EGFR-mutated NSCLC patients who carried clinically stable CNS metastasis.

Interestingly, although no significant difference in PFS was observed between lower dose erlotinib and standard dose gefitinib, duration of response (DOR) with gefitinib 250 mg/d was significantly longer than with erlotinib 100 mg/d. Consistent results were observed in another study. Yamada et al. (18) treated patients with erlotinib 50 mg/d, and then escalated the dose to 150 mg/d in patients with no response. They found that patients having progressive disease at 50 mg/d did not obtain any response when the dose was increased to 150 mg/d. While four patients who had shown tumor shrinkage at 50 mg/d erlotinib achieved partial response with increased dose. These findings indicate that pharmacokinetic factors caused by dose modification may play a greater role in treatment-sensitive clones, but little in resistant clones. Consistently, Foo et al. (22) reported that erlotinib at 150 mg/d failed to substantially inhibit tumors with preexisting T790M clones. Therefore, as long as the administered dose is sufficiently potent in suppressing sensitive clones, disease control and PFS of treatment would not be significantly influenced by dose reduction, as demonstrated in the present study. For patients with responsive tumors, erlotinib 100 mg/d is of ample efficacy, while increasing the dose to 150 mg/d only led to increased toxicity but few incremental efficacies.

By demonstrating the comparable efficacy between lower dose erlotinib and standard dose gefitinib in EGFR-mutated NSCLC, our results could facilitate the development of EGFR TKI-based combination therapies. For example, c-Met amplification has been established as a resistant mechanism to EGFR-TKIs. The combination of erlotinib and crizotinib led to a marked tumor shrinkage (> 50%) in a patient with EGFR-mutant and c-Met-amplified lung adenocarcinoma (25). However, the combination also caused intolerable toxicity that forced a dose reduction to erlotinib 75 mg/d and crizotinib 250 mg/d. The combination of erlotinib 150 mg/d with bevacizumab, ramucirumab, nivolumab, or cabozantinib were also investigated in other studies, where increased efficacy and toxicity were reported for the combination therapy (9–11, 26, 27). Results of the present study indicate the alternative role of lower dose erlotinib in combination therapies, which could lead to comparable efficacy and improved tolerability.

There are some limitations of the current study. First, the recruitment took 5 years to complete because of several competitive trials were initiated during this time. The approval of osimertinib in China further affected the enrollment of this study, because many patients preferred osimertinib over first-generation TKIs. Second, we were unable to evaluate serum concentrations of erlotinib administered at 100 mg/d in this study due to the lack pharmacokinetic data. Finally, study sample size was calculated with DCR as the primary endpoint and the number of participants enrolled in the erlotinib arm was < prespecified 80 participants. A total of 157 patients may not be large enough to tell the mild difference in PFS between the two groups. Only a non-significant trend toward improved tolerability was observed with lower dose erlotinib, which could also be attributed to the small sample size. Future studies with larger sample size are warranted to expand on our findings.

In conclusion, this study provided the first RCT-based evidence on efficacy and tolerability of 100 mg erlotinib in EGFR-mutated, advanced NSCLC. Compared with gefitinib at 250 mg/d, erlotinib at 100 mg/d yielded comparable efficacy in terms of disease control, tumor response, median PFS, and median OS. Similar results were also observed in patients in the first-line setting, patients with different EGFR mutations and patients with or without baseline CNS metastasis. Therefore, in Stage IV EGFR mutated NSCLC, this study showed that erlotinib 100 mg/d had similar DCR compared with gefitinib 250 mg/d. A randomized phase-3 non-inferiority trial with PFS as a primary endpoint is required to confirm the non-inferiority of erlotinib 100 mg/d when compared with gefitinib 250 mg/d.

## Data Availability Statement

The raw data supporting the conclusions of this article will be made available by the authors, without undue reservation.

## Ethics Statement

The studies involving human participants were reviewed and approved by Sun Yat-sen University Cancer Center. The patients/participants provided their written informed consent to participate in this study.

## Author Contributions

LZ, SZ, ZheZ, and WF contributed to study design, data collection, data interpretation, and drafting of the manuscript. LZ supervised the study. SZ, ZheZ, WF, YZ, ZhoZ, and SH contributed to data collection and management. All authors were involved in the provision of study materials and patients, data interpretation, contributed to the writing, and critical review of the manuscript.

## Conflict of Interest

LZ has received research support from Qilu Pharmaceutical Co. Ltd, AstraZeneca, Eli Lilly, Roche, and Bristol-Myers Squibb. The remaining authors declare that the research was conducted in the absence of any commercial or financial relationships that could be construed as a potential conflict of interest.
